# Elevated Levels of miR-155 in Blood and Urine from Patients with Nephrolithiasis

**DOI:** 10.1155/2014/295651

**Published:** 2014-08-14

**Authors:** Yang-Yang Hu, Wei-Da Dong, Yun-Fei Xu, Xu-Dong Yao, Bo Peng, Min Liu, Jun-Hua Zheng

**Affiliations:** ^1^Department of Urology, Shanghai Tenth People's Hospital, Tongji University, Shanghai 200072, China; ^2^Department of Otolaryngology, First Affiliated Hospital of Nanjing Medical University, Nanjing, Jiangsu 210029, China

## Abstract

*Background.* Both circulating and urinary miRNAs may represent a potential noninvasive molecular biomarker capable of predicting chronic kidney disease, and, in the present study, we will investigate the serum and urinary levels of miR-155 in patients with nephrolithiasis. *Methods.* Serum and urinary levels of miR-155 are quantified in 60 patients with nephrolithiasis; the result was compared to 50 healthy volunteers. Estimated glomerular filtration (eGFR) was calculated and, by simple regression analysis, the correlations of miR-155/eGFR and miR-155/CRP (C-reactive protein) levels were analyzed as well. *Results.* The median levels of serum and urinary levels of miR-155 are significantly higher in nephrolithiasis patients than in controls. eGFR inversely correlates with urinary level of miR-155; CRP positively correlates with urinary miR-155. Urinary level of miR-155 inversely correlates with urinary expression of interleukin- (IL-) 1*β*, IL-6, and tumor necrosis factor- (TNF-) *α* and positively correlates with urinary expression of regulated upon activation, normal T-cell expressed, and secreted (RANTES). *Conclusion.* Serum and urinary levels of miR-155 were significantly elevated in patients with nephrolithiasis, and the upregulation of miR-155 was correlated with decline of eGFR and elevation of CRP. Our results suggested that miR-155 might play important roles in the pathophysiology of nephrolithiasis via regulating inflammatory cytokines expression. Further study on the molecular pathogenic mechanism and larger scale of clinical trial are required.

## 1. Introduction

Nephrolithiasis is a condition involving the development of stones in the kidney; it is a common disease with a prevalence of 5–8% worldwide [1]. The exact cause and etiology of nephrolithiasis remained unclear. The risk factors for developing nephrolithiasis include genetics, age, sex, geography, seasonal factors, diet, and occupations [2]. No specific predictive biomarker for the disease come on the scene and many patients are diagnosed late after marked symptoms such as renal colic and hematuria appear. A reliable biomarker for nephrolithiasis which could predict earlier diagnosis, treatment, or better monitoring is greatly demanded.

The underlying mechanisms of immune response activation in the etiology of nephrolithiasis have been recently proposed however not clarified [3]. MicroRNAs (miRNAs) are noncoding, single-stranded RNA molecules of 21 to 23 nucleotides in length; miRNAs regulate gene expression at posttranscriptional level by degrading or blocking translation of messenger RNA (mRNA) and play important roles in many physiological and pathological processes [4, 5]. A number of miRNA species, notably miR-155, have been shown to regulate multiple steps in the development and function of lymphocytes and myeloid cell [6, 7].

The role of miRNA in the pathogenesis of nephrolithiasis remains as yet unelucidated. A previous study that used miRNA microarray technology revealed intrarenal dysregulation of a number of miRNA species in patients with nephrolithiasis [8]. Our previous studies showed that intrarenal levels of proteins related to epithelial-mesenchymal transition (EMT), such as TWIST and E-cadherin, were diversely regulated and correlated with disease severity and deterioration of renal function in patients with nephrolithiasis [9], and literatures reported that urinary level of EMT related miRNA, such as miR-200a, miR-200b, and miR-429, was reduced and the degree of reduction correlated with disease severity in progression of chronic kidney disease [10]. To date, there were no studies that have examined in detail the urinary miRNAs profiles in the patients of nephrolithiasis. In the present study, we will compare the serum and urinary levels of immune-related miRNA-155 (miR-155) levels between the patients with nephrolithiasis and healthy controls.

## 2. Materials and Methods

### 2.1. Patients

We conducted a case control study between July 2011 and September 2012 in Tongji University Tenth Peoples' Hospital. Patients who were diagnosed as nephrolithiasis during the period were enrolled in this study. Briefly, they were diagnosed by ultrasonography and radiography. No case was found by X-ray to have radiolucent stones or by clinical evaluation to have cystine or uric acid stones. If stone specimens were removed by surgery or obtained after medical treatment or shock-wave lithotripsy, composition of the stones was confirmed by infrared spectroscopy (Spectrum RX I Fourier Transform-Infrared System, Perkin-Elmer, USA) [11]. All patients were followed every two months for at least 12 months. And renal function was assessed at every visit. Clinical data include serum creatinine and urine routine was determined by the responsible physicians and not affected by the study. All physicians were blinded from the results of miRNA measurements.

Normal controls were randomly selected from subjects receiving general health examinations at the same hospital during the same period. The controls had no past history of nephrolithiasis and no clinical findings of stones, which was confirmed by plain abdominal X-ray and abdominal ultrasound. Both cases and controls were excluded if they had a history of chronic urinary tract infection, renal failure, chronic diarrhea, gout, renal tubular acidosis, autoimmune diseases, primary and secondary hyperparathyroidism, and cancer. And they were excluded if they had history of inflammatory disorders and recent inflammations. All study subjects were living in Shanghai. The study was approved by the Institutional Review Board of Tongji University Shanghai Tenth People's Hospital, and written informed consent was obtained from all participants.

### 2.2. Samples Collection and Assays

Since literature reported that staghorn nephrolithiasis was usually correlated with decline of estimated glomerular filtration (eGFR), we measured overfasting serum levels of creatinine (Cr) in each patients (Wako Pure Chemical Industries, Osaka, Japan) and the eGFR was calculated by the equation [12]:

(1) eGFR(mL/min⁡/1.73 m2)=194×Cr−1.094×age−0.287                        in  males,eGFR(mL/min⁡/1.73 m2)=194×Cr−1.094×age−0.287×0.739                            in  females.

Whole blood samples (3 mL) were collected in admission and processed for the isolation of serum within 4 h of collection. The blood was centrifuged at 2000 g for 10 min at room temperature. Serum was then transferred to RNase-free tubes and stored at −80°C.

Urine specimen was collected and processed immediately. Urine sample was centrifuged at 3000 g for 30 minutes and at 13000 g for 5 minutes at 4°C. Supernatant was discarded and the urinary cell pellet was lysed by RNA lysis buffer (Qiagen Inc., Ontario, Canada). Specimens were then stored at −80°C until use.

RNA was isolated by using a Trizol-based miRNA isolation protocol in which 300 *μ*L serum was mixed with 1200 *μ*L Trizol (Invitrogen, USA). After the addition of Trizol, we supplemented the serum with 2 *μ*L synthetic cel-lin-4 (Shanghai GenePharma, China) and subsequently mixed it with 200 *μ*L chloroform. The organic and aqueous phase was separated by centrifugation. The aqueous phase containing the RNA was carefully removed, and RNA was precipitated as described previously [13]. RNA was solubilized by the addition of 25 *μ*L RNase-free water.

TaqMan microRNA Reverse Transcription Kit (Applied Biosystems, Foster City, CA, USA) and High Capacity cDNA Reverse Transcription Kit (Applied Biosystems, Foster City, CA, USA) were used for reverse transcription. For miRNA, 0.5 *μ*g total RNA was mixed with 1 *μ*L specific primers, 0.05 *μ*L 100 mM dNTPs (with dTTP), 0.5 *μ*L 10x reverse transcription buffer, 0.33 *μ*L (50 U) MultiScribe Reverse Transcriptase, and 0.66 *μ*L RNase inhibitor (20 U/*μ*L) and made up to 5 *μ*L with H_2_O. Reverse transcription was performed at 16°C for 30 minutes, 42°C for 30 minutes, and 85°C for 5 minutes. For mRNA, 3 *μ*g total RNA was mixed with 2 *μ*L specific primers, 0.8 *μ*L 100 mM dNTPs (with dTTP), 2 *μ*L 10x reverse transcription buffer, 1 *μ*L (50 U) MultiScribe Reverse Transcriptase, 1 *μ*L RNase inhibitor (20 U/*μ*L) and made up to 20 *μ*L with H_2_O. Reverse transcription was performed at 25°C for 10 minutes, 37°C for 120 minutes, and 85°C for 5 minutes.

Serum and urinary levels of miR-155, together with urinary mRNA of interleukin-1*β* (IL-1*β*), interleukin-6 (IL-6), tumor necrosis factor-*α* (TNF-*α*), and regulated upon activation, normal T-cell expressed, and secreted (RANTES), were quantified by RT-QPCR using the ABI Prism 7900 Sequence Detection System (Applied Biosystems, Foster City, CA, USA). Commercially available TaqMan primers and probes, including 2 unlabeled PCR primers and 1 FAMTM dye-labeled TaqMan MGB probe, were used for all the targets (all from Applied Biosystems). For mRNA expression, the primer and probe set was deliberately designed across the intron-exon boundary so as not to detect probable genomic DNA. For RT-QPCR, 2.5 *μ*L universal master mix, 0.25 *μ*L primer and probe set, 0.33 *μ*L cDNA, and 1.92 *μ*L H_2_O were mixed to make a 5 *μ*L reaction volume. Each sample was run in triplicate. RT-QPCR was performed at 50°C for 2 minutes and 95°C for 10 minutes, followed by 40 cycles at 95°C for 15 seconds and 60°C for 1 minute. *β*-Glucuronidase (GUSB, Applied Biosystems) and RNU48 (Applied Biosystems) were used as house-keeping genes to normalize the mRNA and miRNA expression, respectively [14, 15]. Results were analyzed with Sequence Detection Software version 2.3 (Applied Biosystems). In order to calculate the differences of expression level for each target among samples, the ^ΔΔ^CT method for relative quantitation was used. Average expression level of normal blood samples and urine from normal subjects was used as calibrator for serum and urinary expression and the expression level of targets was a ratio relative to that of the controls.

### 2.3. Statistical Analysis

All the results were presented in mean ± standard deviation for data normally distributed and median and interquartile range (IQR) for the others. Since data of gene expression levels were highly skewed, either log transformation or nonparametric statistical methods were used. We used Mann-Whitney *U* test to compare gene expression levels between groups and Spearman's rank-order correlations to test associations between gene expression levels and clinical parameters. SPSS software (Version 17.0, SPSS Inc., Chicago, IL, USA) was used for statistical analyses and *P* < 0.05 was considered statistically significant.

## 3. Results

This study included 60 nephrolithiasis patients and 50 normal healthy people in the control group. The mean (±SD) of age of patient in the study and control groups was 46.5 ± 14.2 and 44.6 ± 12.8 years, respectively. The ratio of male/female was 37/23 in the study group and 35/15 in the control group. The demographic and baseline clinical data of both groups are summarized in Table 1. There were no significant differences in these parameters between patients and control. Calcium oxalate and calcium phosphate were most prevalent in the nephrolithiasis patients (83%). Urine characteristics of both groups, including urine volume, PH, and uric acid, were listed in Table 1.

### 3.1. Levels of miRNA-155 in Serum and Urine

To date, there is no suitable reference miRNA available for normalization of expression in urine supernatant. Mostly, small RNAU6 (RNU6B) and RNU48 were used as reference genes when voided urine was analyzed, but neither was regularly detected in urine supernatant. Only the study by Hanke et al. reports individual miRNA-miR-152 detection in all urine samples and its use as an endogenous control [16]. So, we used median and interquartile range (IQR) for the others to quantify the expression profile of miRNA-155.

**Figure 1:**
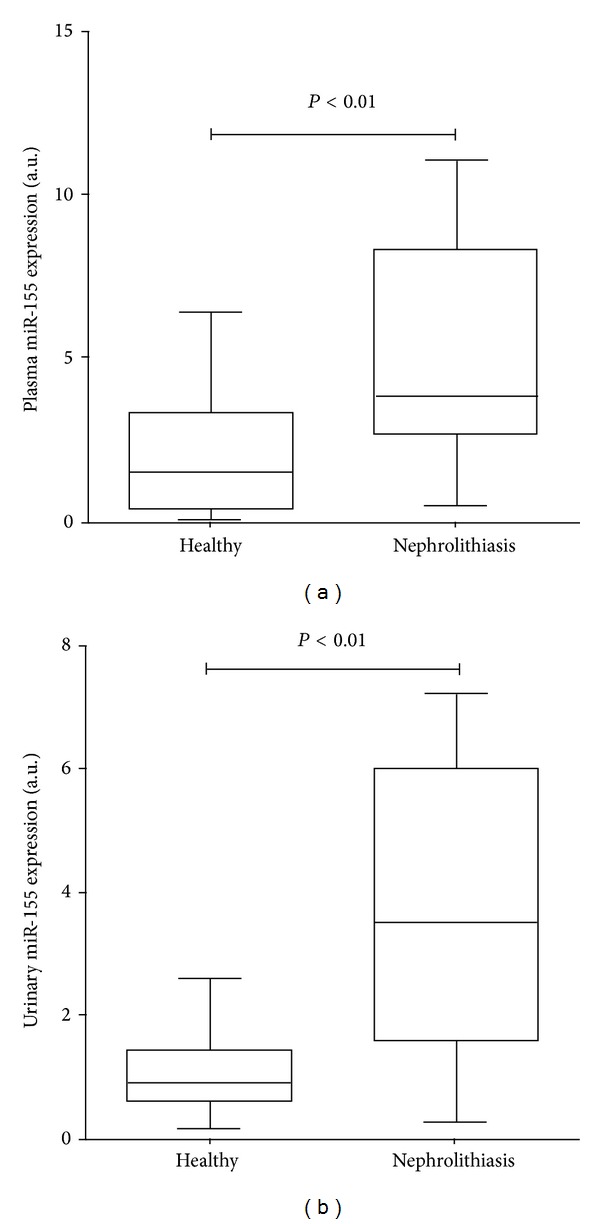
Comparison of serum (a) and urinary (b) levels of miR-155 between patients with nephrolithiasis and normal controls. Data are compared by Mann-Whitney *U* test. Levels are represented as ratio to the average of controls.

As compared to healthy control, the serum miR-155 levels were significantly higher in nephrolithiasis patients (3.83, IQR from 2.55 to 8.29) versus 1.67, IQR from 0.43 to 3.24, *P* < 0.01 (Figure 1(a)). Similarly, nephrolithiasis patients group had a significantly higher urinary levels of miR-155 (3.36, IQR from 1.69 to 6.02 versus 0.99, IQR from 0.76 to 1.44, *P* < 0.01) than healthy cohorts (Figure 1(b)).

### 3.2. Correlations between Urinary miR-155 Levels and Clinical Parameters

**Figure 2:**
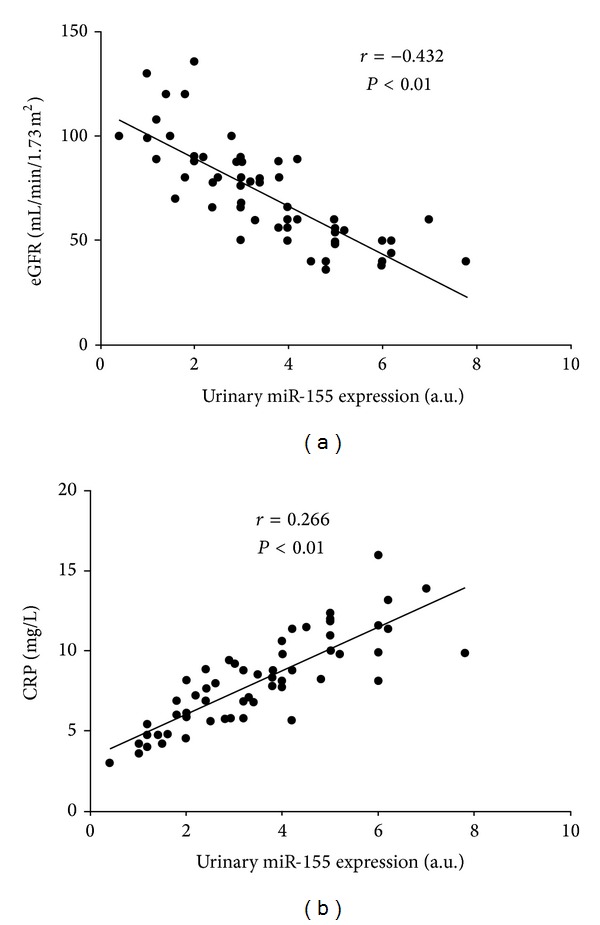
Relation between urinary levels of miR-155 and (a) estimated glomerular filtration (eGFR) and (b) CRP. Data are compared by Spearman's rank correlation coefficient.

Since only 30 healthy controls provided us with the serum samples for detection of miR-155, however, all patients and healthy controls donated urine samples for study; it seems that the detection of urine samples is noninvasive and more popular and acceptable by subjects. We analyzed correlations between urinary miR-155 levels and clinical parameters including C-reactive protein, estimated glomerular filtration (eGFR). Urinary levels of miR-155 significantly and inversely correlate with the eGFR (*r* = −0.432, *P* < 0.01), and urinary levels of miR-155 positively correlate with serum CRP (*r* = 0.266, *P* < 0.01); the details were shown in Figure 2.

### 3.3. Correlations between Urinary miR-155 Levels and Urinary Cytokine Gene Expression

**Figure 3:**
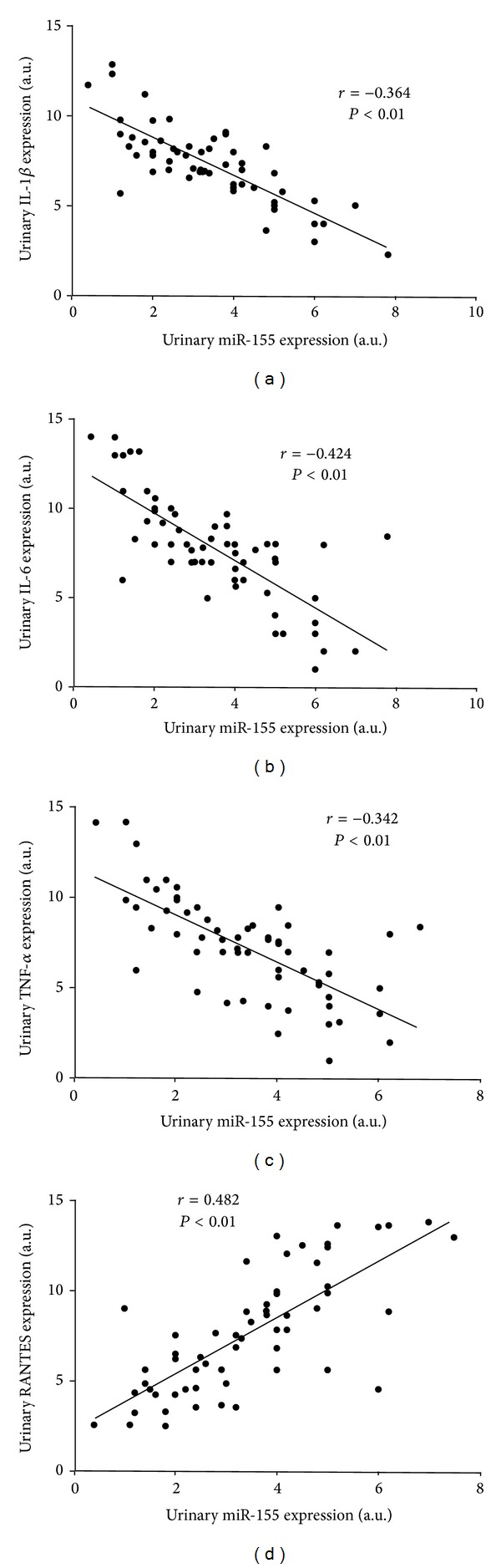
Relation between the urinary levels of miR-155 and (a) IL-1*β*, (b) IL-6, (c) TNF-*α*, and (d) RANTES. Data are compared by Spearman's rank correlation coefficient.

By simple regression analysis of the parameters, we found that urinary levels of miR-155 inversly correlate with urinary expression of IL-1*β*, IL-6, and TNF-*α*, and positively correlate with urinary expression of RANTES (Figure 3).

## 4. Discussion

Our study investigated the serum and urinary levels of miR-155 in nephrolithiasis patients and initially found that the miR-155 expressions in the patients with nephrolithiasis were significantly higher than those in normal subjects. The miR-155 levels inversely correlate with the eGFR and positively correlate with serum CRP. In addition, by a simple regression analysis, we demonstrated that urinary miR-155 levels significantly and positively correlated with urinary expression of RANTES and significantly and negatively correlated with IL-1*β*, IL-6, and TNF-*α*, which suggests the potential inflammatory pathways involved in etiology of human nephrolithiasis.

In the past studies, it has been reported that multiple miRNAs play important roles in the pathogenesis of a variety of kidney diseases. For instances, a panel of urine miRNAs was identified as potential biomarkers for monitoring graft function and anticipating progression to chronic allograft dysfunction (CAD) in kidney transplant [17]. miR-15a and miR-17 are linked to polycystic kidney disease [18, 19] and miR-21, miR-192, miR-216, and miR-377 are linked to diabetic nephropathy [20–23]. To our knowledge, this is the first study suggesting the participation of miR-155 in the pathophysiology of nephrolithiasis. It is already known that miR-155 has been detected in end-stage renal failure patients and there is no clarification of the mechanism of miR-155 in promoting kidney disease progression [24].

In Saikumar et al.'s study, urinary levels of miR-155 could be a useful biomarker in acute kidney injury (AKI) patients. miR-155 was correlated with elevated levels of urinary kidney injury molecule-1 (KIM-1). Similarly, cystatin C, neutrophil gelatinase-associated lipocalin (NGAL), IL-18, and kidney injury molecule-1 (KIM-1) were elevated in ICU patients with prerenal AKI compared to those without AKI [25]. As already known, the etiology of renal calculi is closely related to acute/chronic renal tubular injuries and subsequent crystal deposition and Randall's plaque formation. Furthermore, it has been demonstrated that miR-155 is important in the pathogenesis of chronic kidney disease, including activation of pathways associated with inflammation, fibrosis, extracellular matrix accumulation, and endothelial dysfunction [26]. However, whether miR-155 is elevated or decreased in kidney stone patients, no relative studies emerged until our present investigation.

The role of miR-155 has been well documented in the regulation of the inflammatory response in models of experimental autoimmune encephalomyelitis, graft versus host disease, alcoholic liver disease, and rheumatoid arthritis [27–30]. Considering the fact that ischemia-reperfusion injury is an inflammatory event and miR-155 is involved in the pathogenesis of ischemia-reperfusion injury, we included miR-155 as a candidate in our study [31].

miR-155 regulates the expression of adhesion molecules in inflammatory endothelial cells (ECs) as well as inflammation response in ECs mediated by and angiotensin *α* [32]. Another study revealed that miR-155 and angiotensin *α* type 1 receptor (AT1R) are coexpressed in ECs and vascular smooth-muscle cells and their expression is negatively correlated with the expression of the endogenous AT1R [33]. A silent polymorphism (+1166 A/C) in the human AT1R has been shown to be associated with cardiovascular disease (CVD), which might be mediated by enhanced AT1R activity. Therefore, miR-155 might be responsible for endothelial activation and increased CVD risk [34]. In a latest meta-analysis of cohort study, kidney stones were associated with increased cardiovascular risk. In fact, the association between nephrolithiasis and many chronic kidney diseases suggests a common causative link. There are indications that stone formation can lead to hypertension, diabetes, chronic disease, and myocardial infarct. The reverse also appears to be true in that diabetes and hypertension can lead to stone formation. The production of reactive oxygen species (ROS) and the development of oxidative stress (OS) are common features of many renal and cardiovascular diseases including, hypertension, diabetes, metabolic syndrome, and nephrolithiasis. So, we can hypothesize that oxidative stress produced by one disease may lead to another disease under suitable conditions. And also, in a recent study, Tian et al. reported a new regulatory pathway of YY1/HDACs/miR-155/HBP1 in macrophage-derived foam cell formation during early atherogenesis; in this study, the potential role of miR-155 in mediating oxLDL-induced lipid uptake and reactive oxygen species (ROS) production of macrophages is demonstrated [35].

Overwhelming evidence has proved the involvement of miR-155 in the regulation of both innate and adaptive immune responses [4, 5]. Given the importance of renal immune response in the pathophysiology of kidney stones, we investigated the correlations between miR-155 and important proinflammatory cytokines: IL-1*β*, IL-6, and TNF-*α*, a chemokine (RANTES) in the urine sediment. In line with the previous studies which showed that both miR-146a and miR-155 exerted a regulation activity to limit the overproduction of proinflammatory cytokines [36], we found urinary levels of miR-155 negatively correlated with the expression of IL-1*β*, IL-6, and TNF-*α* in patients with nephrolithiasis. Our result supports the hypothesis that the upregulation of miR-155 results in suppression of IL-1*β* and IL-6 production. On the contrary, we found that urinary levels of miR-155 positively correlated with RANTES. The effect of miR-155 on RANTES expression and function in nephrolithiasis requires further study.

There are a few limitations of our study. First, it was a relatively small retrospective biomarker qualification study with 60 nephrolithiasis patients. Larger studies will be needed to confirm the expression profiles of miR-155 in this population. Second, we detected the levels of miR-155 using whole blood and urine sediment without determining the cellular sources for each of them. Since miR-155 is related to immune response, it may be expressed in multiple cell types, and future studies would be necessary to investigate its expression level in specific cell type. Thirdly, we did not conduct a function study of miR-155. The underlying mechanism of the changes and correlations observed in this study needs further investigation. Finally, the present study is only cross-sectional in design, and it is possible that miR-155 levels alter with disease progression and therapy. Whether there is correlation between nephrolithiasis size, position, and miR-155 expression level, future studies are needed.

In conclusion, we found that both serum and urinary levels of miR-155 are upregulated in nephrolithiasis patients. The investigation of miR-155 suggests that urologists should be concerned about previous neglected renal function deterioration in clinical kidney stone patients and it greatly enhances our understanding of tubular injury in etiology of nephrolithiasis. Further validation of various inflammatory cytokines implies that the urinary levels of miR-155 correlated with inflammatory events in nephrolithiasis cohort, which suggested that miR-155 might play an important role in the pathophysiology of human nephrolithiasis and further study is needed.

## Figures and Tables

**Table 1:** General characteristics of nephrolithiasis patients and healthy controls.

Factor	Nephrolithiasis (*n* = 60)	Healthy controls (*n* = 50)
Age (years)	46.5 ± 14.2	44.6 ± 12.8
Gender (male/female)	37/23	35/15
Body weight (kg)	61.65 ± 10.86	64.84 ± 10.52
Height (m)	1.69 ± 0.09	1.71 ± 0.08
Body mass index (kg/m^2^)	22.45 ± 5.88	23.58 ± 4.36
Urine volume (mL/24 h)	1456 ± 530.8	1162 ± 495.6
Specific gravity	1020 ± 7.6	1022 ± 8.2
Urine creatinine (g/24 h)	1.7 ± 0.7	1.6 ± 0.8
Urine pH	6.20 ± 0.46	6.12 ± 0.68
Urine uric acid (mg/24 h)	665 ± 152	678 ± 140
Blood pressure		
Systolic (mmHg)	140 ± 11.58*	126 ± 9.6
Diastolic (mmHg)	85.3 ± 10.5*	80.6 ± 8.4
eGFR (mL/min/1.73 m^2^)	87.6 ± 44.36	—
CRP (mg/L)	10.12 ± 6.15*	3.2 ± 1.48

*P < 0.01 patients compared with healthy controls.

## Abbreviations

miRNAs: MicroRNAs
CRP: C-reactive protein
EMT: Epithelial-mesenchymal transition
eGFR: Estimated glomerular filtration
TNF: Tumor necrosis factor
RANTES: Regulated upon activation, normal T-cell expressed, and secreted
Cr: Creatinine
IL: Interleukin
IQR: Interquartile range
CAD: Chronic allograft dysfunction
AKI: Acute kidney injury
CKD: Chronic kidney disease
KIM: Kidney injury molecule
ICU: Intensive care unit
AT1R: Angiotensin *α* type 1 receptor
CVD: Cardiovascular disease
ROS: Reactive oxygen species
OS: Oxidative stress.
